# Enhanced autophagy interacting proteins negatively correlated with the activation of apoptosis-related caspase family proteins after focal ischemic stroke of young rats

**DOI:** 10.1186/s12868-022-00740-w

**Published:** 2022-09-28

**Authors:** Jie Wang, Zihao Xia, Peng Sheng, Mengmeng Shen, Lidong Ding, Dezhi Liu, Bing Chun Yan

**Affiliations:** 1 Medical College, Institute of Translational Medicine, Department of Neurology, Affiliated Hospital of Yangzhou University, Jiangsu Key Laboratory of Integrated Traditional Chinese and Western Medicine for Prevention and Treatment of Senile Diseases, The Key Laboratory of Syndrome Differentiation and Treatment of Gastric Cancer of the State Administration of Traditional Chinese Medicine, Yangzhou University, Yangzhou, 225001 People’s Republic of China; 2 Department of Peripheral Vascular Surgery, Shuguang Hospital Affiliated to Shanghai University of Traditional Chinese Medicine, Shanghai, 201203 People’s Republic of China; 3 Department of Neurology, Taizhou Second People’s Hospital, Taizhou, 225500 People’s Republic of China; 4 Department of Neurology, Shuguang Hospital Affiliated to Shanghai University of Traditional Chinese Medicine, No.528 Zhang-Heng Road, Pu-Dong New Area, Shanghai, 201203 People’s Republic of China; 5 Jiangsu Key Laboratory of Zoonosis, Jiangsu Co-Innovation Center for Prevention and Control of Important Animal Infectious Diseases and Zoonoses, Yangzhou, 225009 People’s Republic of China

**Keywords:** Neuronal injury, Autophagy interacting proteins, Apoptosis-related proteins, Young ischemic stroke

## Abstract

**Background:**

Neuronal injury induced in young rats by cerebral ischemia reperfusion (CIR) is known to differ substantially from that in adult rats. In the present study, we investigated the specific differences in neuronal injury induced by focal CIR between young and adult rats.

**Results:**

2, 3, 5-triphenyl tetrazolium chloride (TTC) staining revealed a gradual increase in the infarct volume of both young and adult rats in accordance with I/R times and was significantly lower in young rats than in adult rats under the same conditions. The number of cells in the cortex showing immunoreactivity for neuronal nuclei (NeuN) gradually decreased in both young and adult rats in accordance with I/R times; these numbers were significantly higher in young rats than in adult rats under the same conditions. Similarly, as the duration of I/R increased, the degree of glial activation in the cortex penumbra region became more severe in both young and adult groups; however, glial activation was significantly lower in the cortex penumbra region of young rats when compared with that in adult rats. In addition, the expression of Beclin-1 was significantly higher in the infarct penumbra of young rats than adult rats and was more frequently co-expressed with neurons. The levels of autophagy-related proteins increased significantly in the penumbra region after I/R in both young and adult groups, this increase was more pronounced in young rats than in adult rats. Following CIR, analysis revealed significantly lower levels of pro-apoptosis-related factors and significantly higher levels of anti-apoptosis-related proteins in the young rats than in adult rats.

**Conclusions:**

Collectively, the present results suggest that the the reduced levels of neuronal death after CIR in young rats were closely related to enhanced levels of autophagy and reduced levels of pro-apoptosis in neurons.

**Supplementary Information:**

The online version contains supplementary material available at 10.1186/s12868-022-00740-w.

## Introduction

Ischemic stroke is one of a family of diseases characterized by the loss of local neurological function due to dysfunctional blood circulation in the brain. For many years, researchers have extensively used animal models of ischemic stroke to study the mechanisms underlying neuronal cell death caused by cerebral ischemia, as well as to explore methodologies for prevention and treatment [1, 2]. Furthermore, most of the previous studies investigating ischemic stroke have focused on elderly adults and have tended to ignore ischemic stroke in children. To address these issues over the past 2 decades, researchers have demonstrated that Mongolian gerbils represent a highly suitable model for studying ischemic stroke. For example, the Mongolian gerbil can partially resist transient global ischemic brain injury by delaying the timing of neuronal death and by reducing the number of neuronal deaths [3]. In addition, preliminary studies from our research group have shown that the survival of neurons in young gerbils after transient global ischemia in gerbils is partially due to the close relationships between increased stress and mechanisms that maintain the levels of calcium-binding proteins, antioxidants, and anti-inflammatory factors [3–5]. However, the specific mechanisms of action underlying these effects following focal ischemic stroke in young rats remain unclear.

Autophagy has attracted increasing levels of interest with regards to its specific role in CIR injury. Furthermore, it has been reported that autophagy may contribute to pro-survival signaling during prenatal cerebral hypoxia and ischemia by inducing activation of the PI3K-AKT-mTOR pathway [6, 7]. In contrast, neuronal death occurs when either autophagy or activity in the PI3K-AKT-mTOR pathway is interrupted. In a previous study, Wang et al. found that phospho-ribosomal transferase-induced autophagy promotes the survival of nerve cells following hypoxia and ischemia [8]. Autophagy levels in the mouse brain are known to gradually decline with age [9, 10]. However, whether the higher levels of autophagy in young animals are associated with neuronal injury induced by cerebral ischemia remain unclear because few studies have investigated changes in autophagy-related signaling pathways following ischemic injury in young animals; the studies that have investigated these changes are thus far incomplete. Therefore, in the present study, we aimed to investigate the the relationship between changes of autophagy and transient focal ischemic injury in young rats using an experimental model of middle cerebral artery occlusion (MCAO).

## Methods and materials

### Experimental animals

The present study used male Sprague–Dawley rats obtained from the Comparative Medicine Center of Yangzhou University (Yangzhou, China). The experiment consisted of a young group (1-month-old Sprague Dawley (SD) rats; n = 210) and an adult group (6-month-old; n = 210). We focused only on the young and adult groups, without including an “old” group. Each group was randomly divided into a sham group (n = 30) and a surgery group (n = 180). Meanwhile, the surgery group was separately divided into 3 sub-groups according to the duration of ischemia (30 min, 60 min and 90 min). Rats were sacrificed after 1 or 3 days of ischemia/reperfusion. The rats were housed in a conventional environment in which temperature (23 °C) and humidity (60%) were both controlled. The rats were held under a 12-h light/12-h dark cycle and had free access to food and water. All procedures involving animals and their care were performed in accordance with current international laws and policies (NIH Guide for the Care and Use of Laboratory Animals, NIH Publication No. 85–23, 1985, revised 1996). All experiments were conducted to minimize the number of animals used and suffering caused. The animal protocol was approved based on ethical procedures and scientific care by the Yangzhou University-Institutional Animal Care and Use Committee (YIACUC-15-0016).

### Middle cerebral artery occlusion (MCAO)

**Fig.1:**
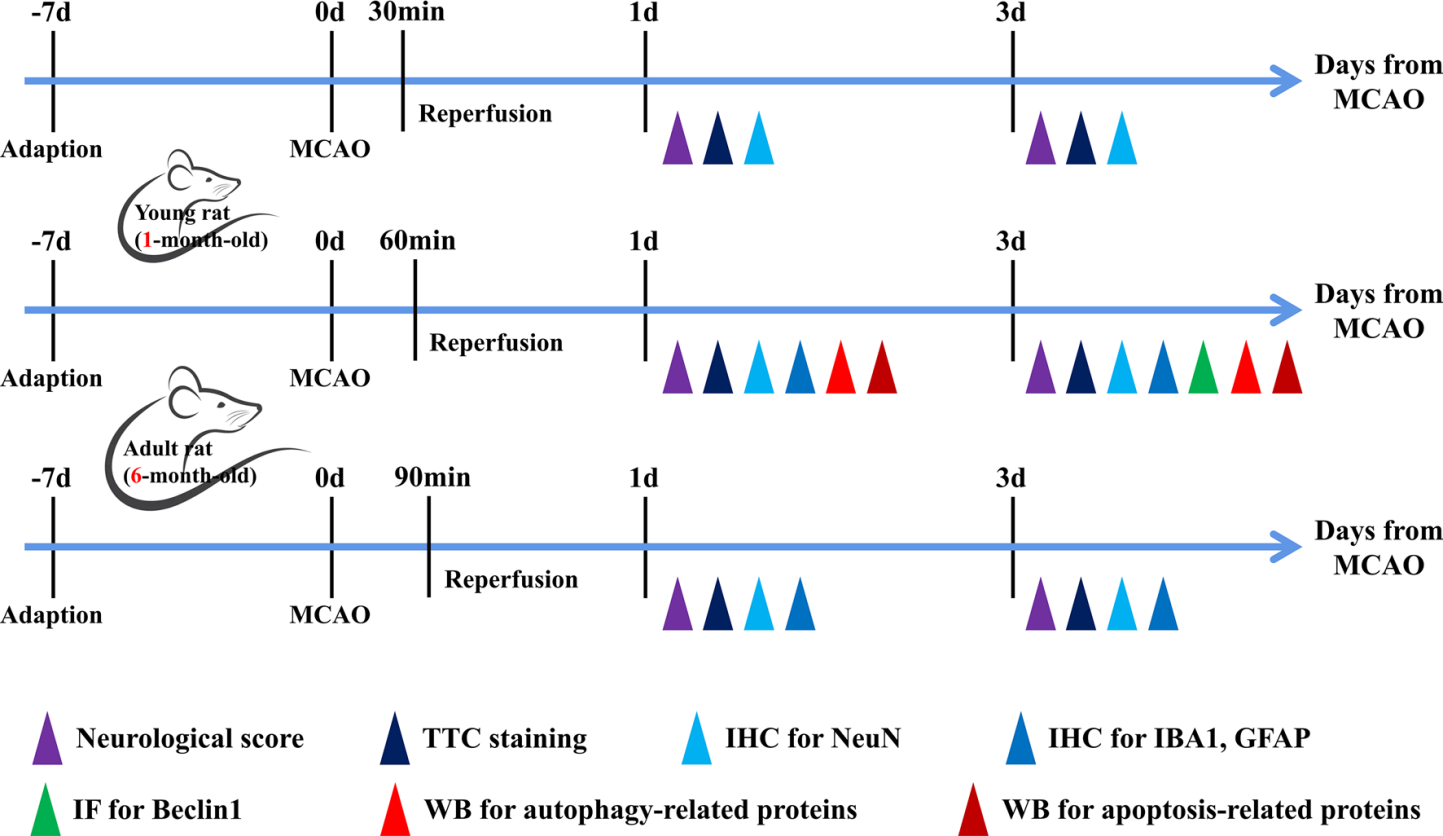
Schematic of experimental protocol

The experimental animals were anesthetized with a mixture of isoflurane (3–4% for induction, 2.5% for maintenance) in 33% oxygen and 67% nitrous oxide via a facemask. After skin preparation and conventional disinfection, the surgical field of view was fully exposed by cutting along the midline of the neck to separate the right common carotid artery, right external carotid artery, right internal carotid artery, and vagus. Ophthalmic scissors were used to make a “V” incision in the external carotid artery, such that it was pulled down and aligned with the internal carotid artery. A nylon monofilament suture (Beijing Cinontech Co. Ltd., Beijing, China) was inserted into the stump of the external carotid artery and then advanced into the lumen of the internal carotid artery until it reached and occluded the middle cerebral artery. The monofilament was removed 30, 60, or 90 min after occlusion to allow reperfusion for 24 or 72 h (Fig. 1); the external carotid artery was then permanently closed. Sham operation groups were subjected to the same surgical procedure, excluding insertion of the monofilament. During surgery, all experimental animals were maintained at a temperature of 37.0–37.9 °C with an automated heating pad connected to a rectal thermometer to avoid rapid reductions in body temperature due to the anesthesia.

### Neurological scores

The neurological deficits of the MCAO and sham rats were assessed with the Bederson and Zea-Longa neurological deficit scores as follows: grade 0: no visible neurological deficits; grade (1) mild neurological deficits, such as dysfunction in stretching the anterior limb; grade (2) unidirectional circling when pulled by the tail; grade (3) rolling movement; grade (4) reduction in consciousness; and grade (5) death. Blinded scoring was performed at the prescribed time.

### 2,3,5-triphenyl tetrazolium chloride staining and the quantification of infarct volume

Following anesthesia, all rats were euthanized with 10% chloral hydrate (Aladdin; Shanghai, China). There brains were then removed and cut into several coronal sections (2 mm thick). The sections were incubated in a 2% solution of 2,3,5-triphenyltetrazolium chloride solution (TTC; Sigma-Aldrich Chemical Co., St. Louis, MO, USA) for 30 min at 37 ℃ and then immersed in a 4% paraformaldehyde solution with phosphate-buffered saline (PBS; pH 7.4) for 6 h. After they had been successfully stained, the sections were photographed and Image-Pro Plus 6 software was used for image analysis. The infarct volume was calculated as the infarct size multiplied by the slice thickness (2 mm) and used to generate the corresponding ratio: (infarct volume/the contralateral non-infarct volume) × 50%.

### Tissue processing for histology

For histological analysis, sham rats (n = 7 at each time point) along with ischemic young (n = 7 at each time point) and adult (n = 7 at each time point) rats were sacrificed at designated times (1 and 3 days after reperfusion). The rats were anesthetized with 2% pentobarbital sodium and then transcardially perfused with 0.1 M PBS (pH 7.4), followed by 4% paraformaldehyde in 0.1 M phosphate buffer (PB; pH 7.4). Next, the brains were removed, post-fixed in the same fixative for 6 h, and then cryoprotected overnight via infiltration with 30% sucrose. Subsequently, the frozen tissues were serially sliced into 30-μm coronal sections on a cryostat (Leica; Wetzlar, Germany); sections were collected in six-well plates containing PBS.

### Immunohistochemistry

To investigate changes in neurons and glial cells following the IR procedure, immunohistochemical staining assays for ionized calcium binding adaptor molecule 1 (Iba1, 1:500, NCNP24,Wako, Japan), glial fibrillary acidic protein (GFAP, 1:800, Chemicon, Temecular, CA), and neuronal nuclei (NeuN, 1:800, Chemicon, Temecula, CA) were carried out with rabbit anti-GFAP (1:1000, Abcam; Cambridge, MA, USA), goat anti-Iba1 (1:1000, Abcam; Cambridge, MA, USA), and rabbit anti-NeuN antibodies (1:1000, CST; Danvers, MA, USA). To establish the specificity of immunostaining, a negative control test was carried out using pre-immune serum, instead of the primary antibodies; this revealed the absence of immunoreactivity in all structures.

Next, 10 sections per animal were selected to quantitatively analyze the immunoreactivity of Iba1, GFAP, and NeuN. We tried to perform tests on equally spaced sections and performed stereological quantification. Digital images of the hippocampal subregions were captured with an image analysis system equipped with a computer-based microscope (Nikon; Tokyo, Japan) and cell counts were obtained by averaging the counts from the sections taken from each animal. In addition, the staining intensities of Iba1-, GFAP-, and NeuN-immunoreactive structures were evaluated based on optical density (OD), which was obtained after the mean gray level had been transformed using the following formula: OD = log (256/mean gray level). Background OD was measured from the areas adjacent to the measured area. After the background density had been subtracted, the ratio of the OD of the image file was calibrated as a percentage (relative OD) using Adobe Photoshop version 8.0 (Adobe, San Jose, CA, USA) and then analyzed using NIH ImageJ 1.59 (NIH, Bethesda, MD, USA). All measurements in each experiment were performed by two observers who were blinded to the experimental conditions to ensure objectivity; experimental samples were assessed under the same conditions.

### Immunofluorescent analyses

Immunofluorescence staining of the brain sections was performed as previously described [11]. In brief, the sections were rinsed twice with PBS, permeabilized with 0.5% Triton X-100 for 10 min, and then blocked with PBS containing 0.2% Triton X-100 and 5% bovine serum albumin for 1 h at room temperature. Next, the sections were incubated with the anti-NeuN (1:1000; Abcam, ab104225) and mouse anti-Beclin-1 primary antibodies (1:400; Santa Cruz Biotechnology; Dallas, TX, USA) in a humidified container for 16 h at 4 ℃. Then, the sections were rinsed twice in PBS and sequentially incubated with the Texas red-conjugated goat anti-mouse-IgG secondary antibody (1:400, Abcam, ab97019) and fluorescein isothiocyanate-conjugated goat anti-mouse-IgG (1:400, Abcam, ab6785) in a humidified lucifugal container for 2 h. Finally, the sections were washed twice in PBS, sealed using SHUR/Mounting medium (Ted Pella Inc.; Redding, CA, USA), and analyzed by fluorescence microscopy (D-Eclipse C1; Nikon).

### Western blot analysis

To investigate protein levels associated with apoptosis and autophagy in the penumbral regions, western blot analyses were performed using samples from the sham, young, and adult groups, which were obtained at 1 and 3 days after ischemic surgery (n = 7 at each time point). Following euthanasia, the brains were removed and serially and transversely cut into a thickness of 400 µm on a vibratome. The designation of the penumbral regions was based on numerous pharmacological and histopathological studies by Ashwal et al. and others that have defined the adjacent ventrolateral cortex as the penumbra [12, 13]. In brief, the brain of each animal was sectioned into 3 slices beginning 2 mm from the anterior tip of the frontal lobe. Section two (4-mm thick) was used for the measurement of western blot analysis. First, a longitudinal cut (from top to bottom) ~ 2 mm from the midline between the 2 hemispheres was made to avoid the mesial hemispheric structures that are supplied primarily by the anterior cerebral artery. Next, the penumbras (adjacent cortex) were separated by a transverse diagonal cut at ~ 30° from the vertical position. The samples were preprocessed with Whole Cell Lysis Assay and Total Protein Extraction kits (KeyGEN; Nanjing, China) and protein concentrations were determined using a Pierce BCA Protein Assay Kit (Thermo Scientific; Rockford, IL, USA).

In brief, equal amounts of protein (30 μg) were separated by 10% sodium dodecyl sulfate polyacrylamide gel electrophoresis and then transferred to nitrocellulose membranes (Millipore; Bedford, MA, USA) for incubation with antibodies; membranes were stripped and re-used as necessary. To reduce background staining, the membranes were then incubated with 5% bovine serum albumin in Tris-buffered saline containing 0.1% Tween 20 for 6 h before incubation with antibodies. According to the molecular weight of target proteins, the membranes were cut into proper bands prior to hybridisation with antibodies. Then, they were immediately incubated with rabbit anti-NeuN (1:1000, CST, #24,307), rabbit anti-SQSTM1/p62 (1:1000, CST, #39,749), rabbit anti-LC3-I/II (1:1000, CST, #2775), mouse anti-Beclin-1 (1:1000, Santa Cruz Biotechnology, SC-48381), rabbit anti-Atg3 (1:1000, CST, #3415), rabbit anti-Atg13 (1:1000, CST, #13,273), rabbit anti-Atg7 (1:1000, CST, #8558), rabbit anti-Caspase-8 (1:1000, CST, #4790), rabbit anti-Caspase-3 (1:1000, CST, #9662), rabbit anti-Bcl-xL (1:1000, CST, #2764), rabbit anti-BAD (1:1000, CST, #9268), rabbit anti-Bcl2 (1:1000, CST, #3498), and rabbit anti-β-actin antibodies (1:1000, CST, #4970) overnight at 4 °C. Subsequently, the membranes were exposed to a goat anti-mouse/rabbit IgG secondary antibody (1:1000, CST, #14,709, #14,708) for 2 h at room temperature and the SuperSignal West Pico Chemiluminescent Substrate (Thermo Scientific) was used for protein detection.

The results of the western blot analyses were scanned and densitometric analysis was conducted using Quantity One Analysis Software (Bio-Rad; Hercules, CA, USA) to quantify the bands. These values were used to determine the relative OD; the ratio of the relative OD was generated as a percentage, with the sham group designated as 100%. Each of the western blots shown herein are representative of at least three similar independent experiments.

### Statistical analysis

Due to accidental sacrifice after surgery, we selected the experimental data of each group (n = 7) for analysis. All experimental data were statistically processed using SPSS 26.0 (IBM Corp., Armonk, NY, USA) and all data are represented as means ± standard errors of the mean. One-way and two-way analysis of variance (ANOVA) were performed to compare normally distributed data. Student–Newman–Keuls and Fisher’s Least Significant Difference tests were used to compare data between groups. *P* values < 0.05 were considered to indicate statistical significance.

## Results

### Differences in neurological function scores between young and adult rats after IR

Following surgery, most adult rats exhibited listlessness, as well as a reduction or lack of eating and drinking. However, young rats were generally more active than adult rats after experiencing the same conditions. There were also significant differences in the neurological function scores of the young and adult rats after IR. Following ischemia for 30 min and 1 day of reperfusion, there was no significant neurological damage among in either the young or adult rats. However, after 3 days of reperfusion, adult rats showed mild neurological impairments (score ≈ 1), while young rats did not. Similarly, following ischemia for 60 min and reperfusion for 1 day, adult rats showed neurological impairments (score ≈ 1), while young rats did not. After 60 min of ischemia and 3 days of reperfusion, young rats showed mild neurological impairments (score ≈ 1) and adult rats showed neurological impairments that were more severe (score ≈ 2).

**Fig. 2:**
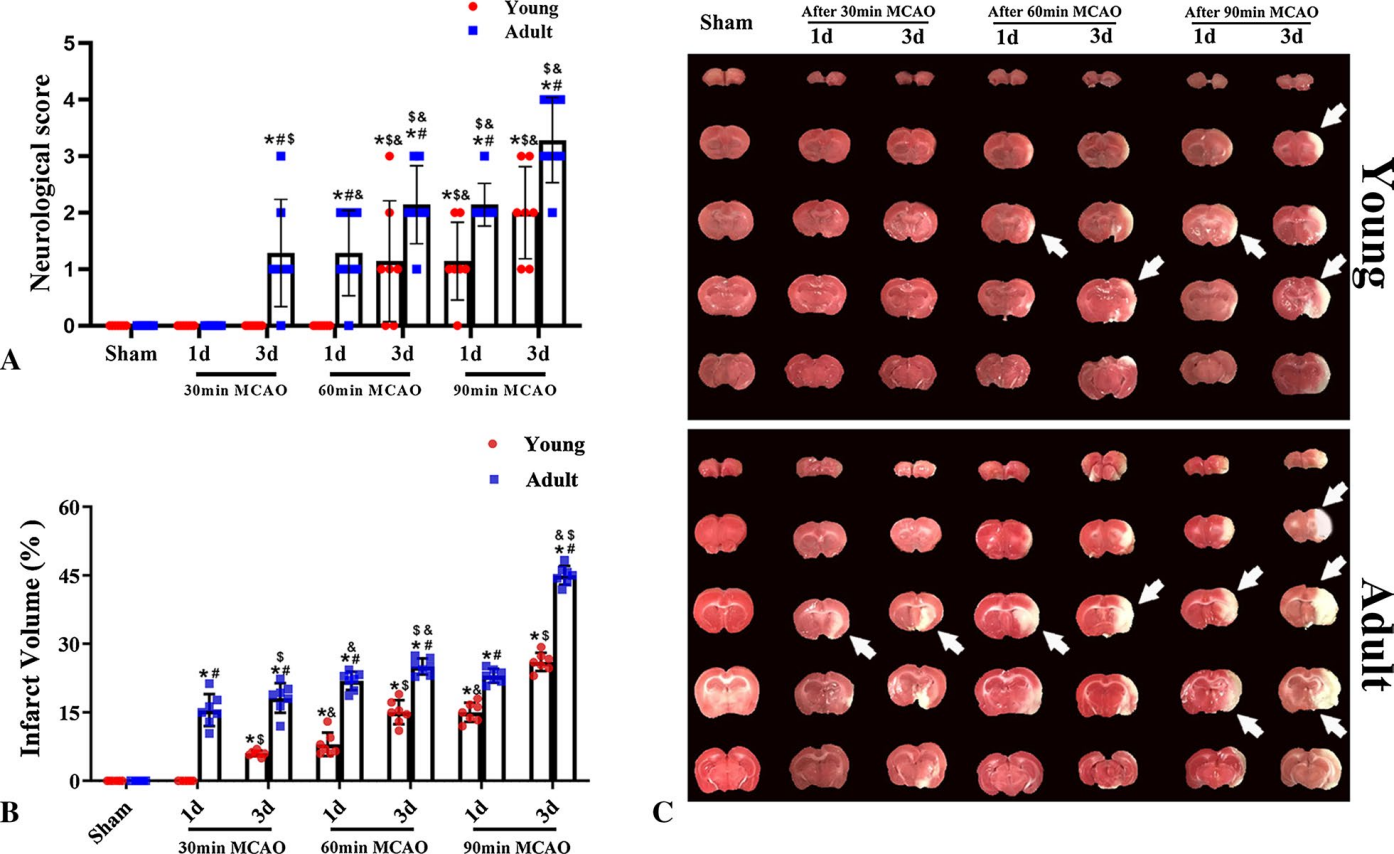
Changes of the neurological function scores and infarct volume between the young and adult groups (1 and 3 days of reperfusion after 30, 60 and 90 min of ischemia). Neurological Scores (**A**) (Specific neurological scores are presented in Additional file 2); The infarct volume was determined with TTC staining, and the white area was the infarct area. The infarct volume was expressed as the ratio of (infarct volume/the contralateral non-infarct volume) × 50% (**B** and **C**). (n = 7 per group; ^*^p < 0.05, significantly different from the respectively sham-group; ^#^*p* < 0.05, significantly different from the corresponding young-group at the same ischemia time; ^$^*p* < 0.05, significantly different from the corresponding 1d group at the same ischemia time; ^&^*p* < 0.05, significantly different from the respectively preceding group at the same reperfusion time.) The bars indicate the means ± SEM

Following 90 min of ischemia and 1 day of reperfusion, the neurological function score of rats in the young group was 1 while that of rats in the adult group was 2. At 3 days after reperfusion, the neurological impairment scores of rats in both the young and adult groups were worse than after 1 day of reperfusion; however, impairments in the young rats were less severe than those of adult rats. Taken together, these results indicate that rats in the young group exhibited significant ability to cope with ischemic injury (Fig. 2A).

### Differences in cerebral infarction volume between young and adult rats after IR

There were also significant differences in cerebral infarction volume between rats in the young and adult groups after IR. On the first day of reperfusion following 30 min of ischemia, there was no significant difference in the cerebral infarction volume between the MCAO and sham groups of young rats, whereas a small infarction volume was observed in the MCAO group of adult rats. After 3 days of reperfusion, the infarction volume in the adult rats was larger, although there was no significant infarction volume in the young rats. Following 60 min of ischemia and 1 day of reperfusion, rats in the adult group showed obvious cerebral infarction foci and the infarction volume was larger after 3 days of reperfusion; the infarct volume was significantly smaller in young group than in adults. Following 90 min of ischemia and 1 day of reperfusion, the infarct volume of rats in the adult group was similar to that observed after 60 min of ischemia and reperfusion for 3 days, in that the infarct volume increased in accordance with reperfusion time. Although the infarct volume of the young group was significantly smaller, it also increased in accordance with reperfusion time. TTC staining results demonstrated that young rats exhibited a significant ability to cope with ischemia when compared to adult rats (Fig. 2B and C).

### Differences in NeuN immunostaining between young and adult rats after IR

Changes in neuronal injury after IR were observed by staining with NeuN, an established neuronal biomarker. Following 30 min of ischemia, there was no obvious neuronal loss or injury in the striatum areas of rats in the young group after either 1 or 3 days of reperfusion. Conversely, there was obvious neuronal loss and injury in adult rats after 3 days of reperfusion. Following 60 min of ischemia and 1 day of reperfusion, there were significant increases in neuronal death in the striatum in adult rats, whereas the injuries and degree of neuronal death in young rats were smaller in young rats. After 3 days of reperfusion, neuronal death was more obvious in adult rats. Although neuronal damage was also more pronounced in young rats, it was less severe than that observed in the adult group.

**Fig. 3:**
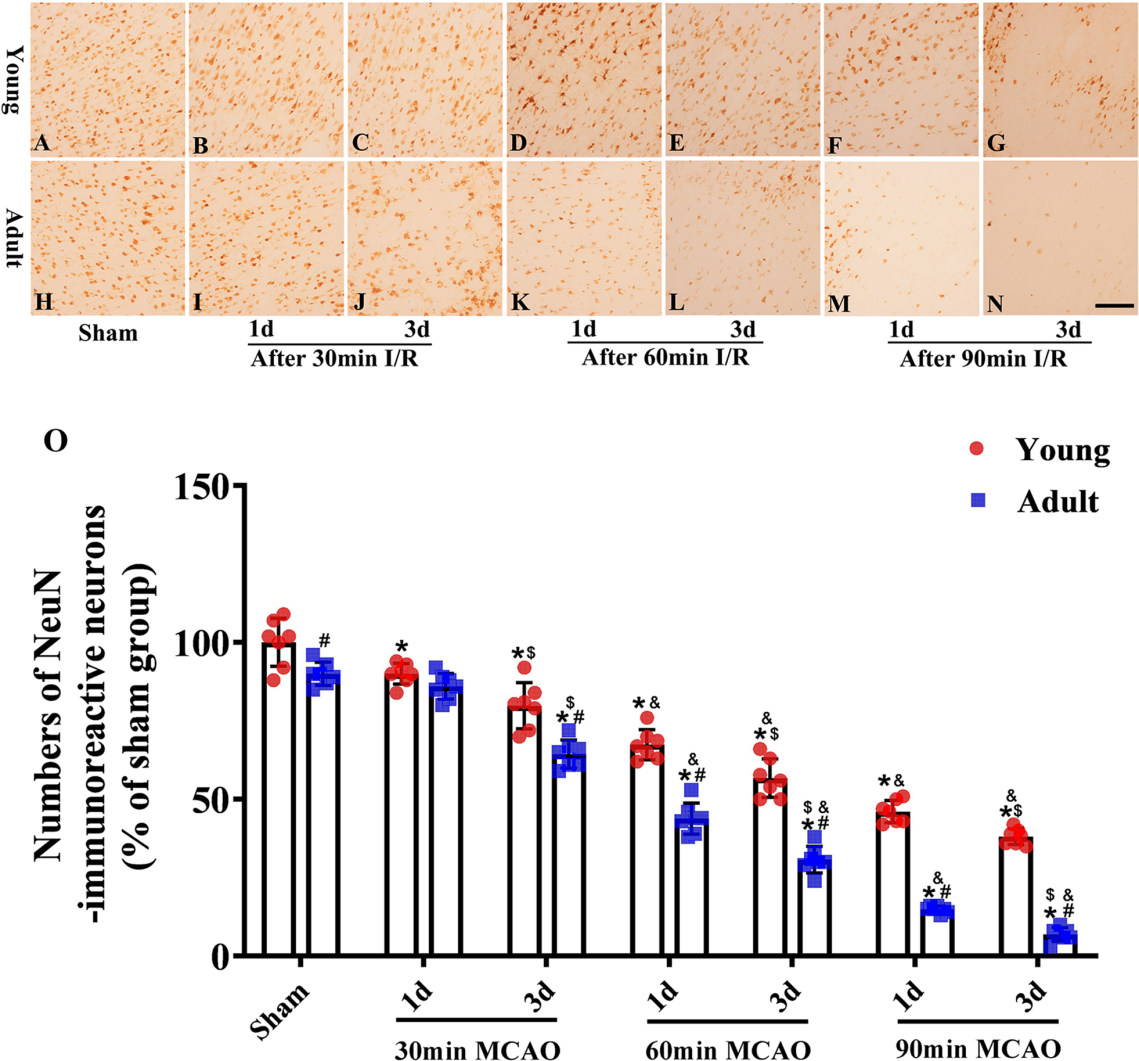
Numbers of NeuN-immunoreactive neurons in the same region of cerebral cortex between the adult (H-N) and young groups (**A**–**G**) (1 and 3 days of reperfusion after 30, 60 and 90 min of ischemia). The numbers of NeuN-immunoreactive neurons (**O**). (n = 7 per group; *p < 0.05, significantly different from the respectively sham-group; ^#^*p* < 0.05, significantly different from the corresponding young-group at the same ischemia time; ^$^*p* < 0.05, significantly different from the corresponding 1d group at the same ischemia time; ^&^*p* < 0.05, significantly different from the respectively preceding group at the same reperfusion time.) The bars indicate the means ± SEM. Scale bars: 100 um (**A**–**N**)

Following 90 min of ischemia, NeuN immunoreactivity around the striatum in adult rats was very weak after 1 day of reperfusion and was almost absent after 3 days of reperfusion; many neurons had died by this timepoint. Although neuronal death was significantly lower in young rats than in adult rats, the degree of neuronal damage and the extent of neuronal death increased in accordance with reperfusion time. NeuN immunoreactivity analyses demonstrated that young rats exhibited a significant ability to cope with ischemia when compared to adult rats (Fig. 3).

Based on these findings, we next investigated the mechanisms underlying the anti-cerebral ischemia effects observed in young rats. Because there were few differences between rats in the young and adult groups after 30 min of ischemia, subsequent experiments focused on ischemia over 60 and 90 min periods.

### Differences in immunoreactive glial cells between young and adult rats after IR

**Fig. 4:**
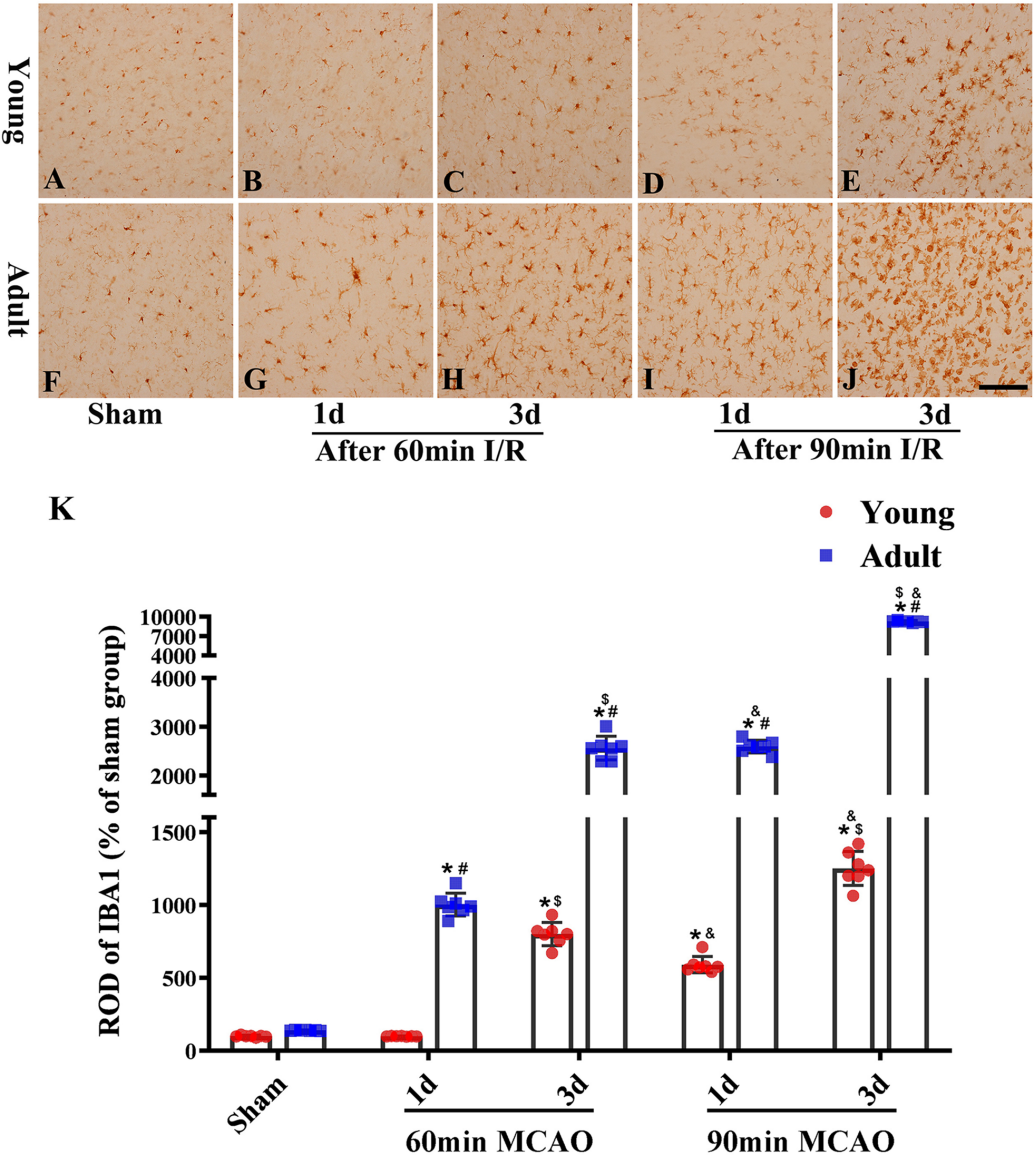
Changes of microglial activation in the same region of cerebral cortex between the adult (**F**–**J**) and young groups (**A**–**E**) (1 and 3 days of reperfusion after 60 and 90 min of ischemia). The ratio of Iba-1 immunoreactivity (**K**). (n = 7 per group; ^*^p < 0.05, significantly different from the respectively sham-group; ^#^*p* < 0.05, significantly different from the corresponding young-group at the same ischemia time; ^$^*p* < 0.05, significantly different from the corresponding 1d group at the same ischemia time; ^&^*p* < 0.05, significantly different from the respectively preceding group at the same reperfusion time.) The bars indicate the means ± SEM. Scale bars: 100 um (**A**–**J**)

**Fig. 5:**
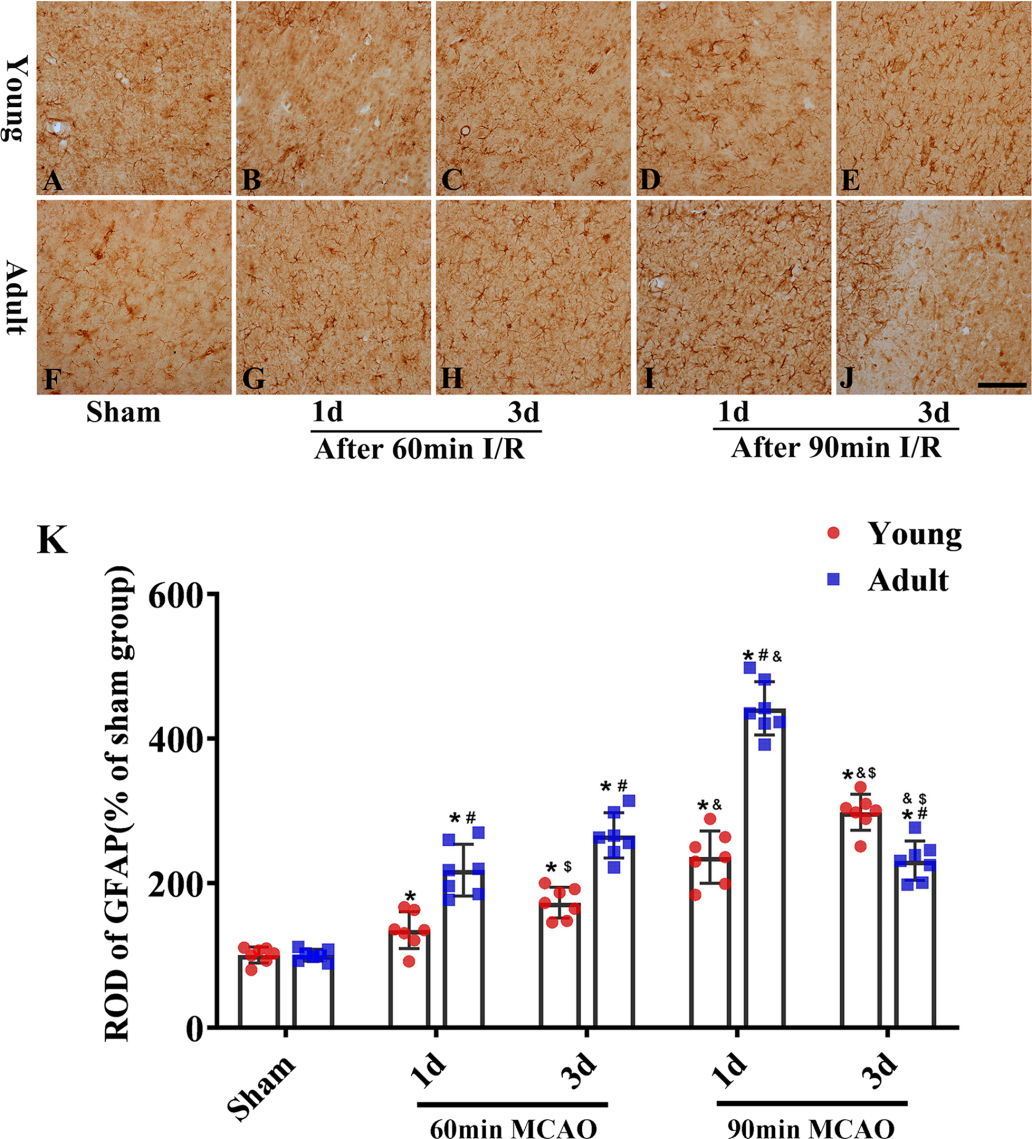
Changes of astrocytic activation in the same region of cerebral cortex between the adult (**F**–**J**) and young groups (**A**–**E**) (1 and 3 days of reperfusion after 60 and 90 min of ischemia). The ratio of GFAP immunoreactivity (**K**). (n = 7 per group; ^*^p < 0.05, significantly different from the respectively sham-group; ^#^*p* < 0.05, significantly different from the corresponding young-group at the same ischemia time; ^$^*p* < 0.05, significantly different from the corresponding 1d group at the same ischemia time; ^&^*p* < 0.05, significantly different from the respectively preceding group at the same reperfusion time.) The bars indicate the means ± SEM. Scale bars: 100 um (**A**–**J**)

Glial cells are involved in the repair and regeneration of neurons after cerebral IR but can also contribute to neuronal death and dysfunction. Therefore, the activations of peri-infarction glial cells (microglial Iba-1 and star glial GFAP levels) were evaluated after cerebral IR injury. Microglial activation occurred after 60 min of ischemia and 3 days of reperfusion in young rats; in contrast, microglial activation began after 1 day of reperfusion among rats in adult rats and was more obvious after 3 days of reperfusion (Fig. 4). Following 90 min of ischemia, microglial cells at the periphery of infarction areas were highly activated in adult rats, but only mildly activated in young rats (Fig. 4). Trends related to the activation of star glial cells were similar to those of microglial cells; activation occurred after 60 min of ischemia and 3 days of reperfusion in young rats but began after 1 day of reperfusion in adult rats and was more obvious after 3 days of reperfusion (Fig. 5). Following 90 min of ischemia, astrocytes at the periphery of the infarctions were highly activated after 1 day of reperfusion in adult rats, although the activation of astrocytes decreased significantly after 3 days of reperfusion. It is possible that this was because most of the astrocytes had been damaged or had died due to severe ischemia injury in adult rats by the third day of reperfusion after 90 min of ischemia (Fig. 5). However, the activation of astrocytes in young rats showed a persistent increase and there was no deficiency in astrocytes when tested on the third day of reperfusion after 90 min of ischemia; this may be due to the increased ability of young rats to cope with cerebral ischemia than adult rats. In addition, the degree of glial cell activation increased in accordance with reperfusion time. Collectively, these results indicated that injuries to nerve cells were alleviated in young rats via the reduced activation of glial cells following ischemia.

### Differences in Beclin-1 fluorescent immunoreactivity between young and adult rats after IR

**Fig. 6:**
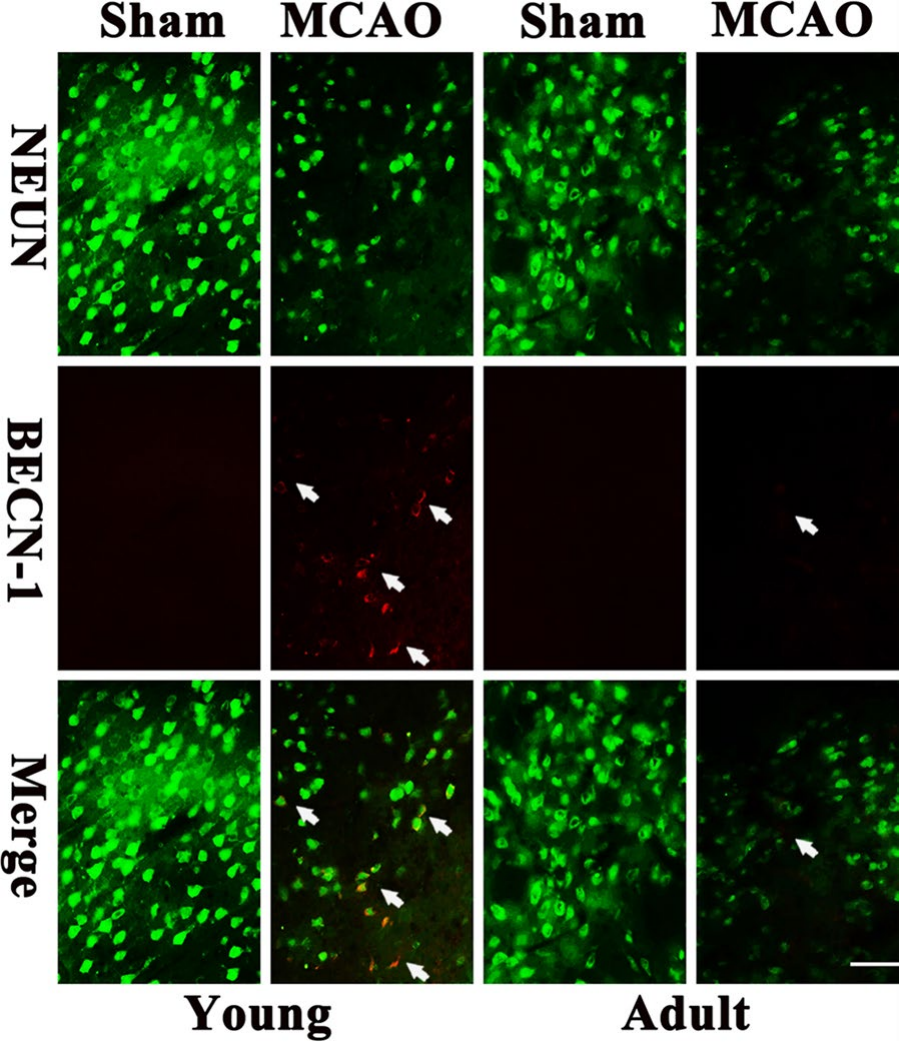
The immunofluorescence of Beclin1 in neurons of penumbral region on the third day reperfusion after 60 min ischemia. The sections were double-labeled with the anti-Beclin1 antibody (red) and anti-NeuN antibody (green). The immunoreactivity of Beclin1 elevated significantly in young group after I/R (n = 7 in each group). Scale bar: 50 µm

Next, we investigated the expression levels of autophagy-related proteins in neurons within the infarcted penumbra after IR. Beclin-1 is regarded as a gatekeeper of autophagy and is often targeted during artificial intervention. Because neuronal injury and death primarily occurred 3 days after IR, we focused on the immune expression of Beclin-1 in the infarcted penumbra following ischemia for 60 min and 3 days of reperfusion. Although there was an absence of Beclin-1 expression in rats from the sham group after 3 days of IR, Beclin-1 immunoreactivity was significantly higher in the penumbra of the infarction in young rats and was co-expressed with neurons. However, there were no significant changes in Beclin-1 expression in the infarcted penumbra region of adult rats and the difference was smaller than that observed in young rats. Collectively, these results indicate that the level of autophagy in neurons after IR increased in young rats and improved IR-induced neuronal death (Fig. 6).

### Changes in autophagy-related proteins between young and adult rats after IR

The expression levels of autophagy-related proteins in the infarcted penumbra were also evaluated after 60 min of ischemia and 3 days of reperfusion. Levels of LC3-II were increased in the adult ischemia-group after 1 and 3 days of reperfusion. However, among rats in the young ischemia-group, the levels of LC3-II were significantly higher than those in the young sham-group. Meanwhile, the increase in the levels of LC3-II after 1 and 3 days of reperfusion was also more pronounced than the adult ischemia-group. Compared to rats in the sham group, the expression levels of p62 were lower in both young and adult rats after 1 and 3 days of reperfusion. In young rats, the expression levels of p62 were generally lower than those of adult rats. In addition, Beclin-1 expression levels were higher in adult rats after 1 and 3 days of reperfusion. In young rats, Beclin-1 expression levels exhibited a significant increase after 3 days of reperfusion and were generally higher than those in adult rats.

**Fig. 7:**
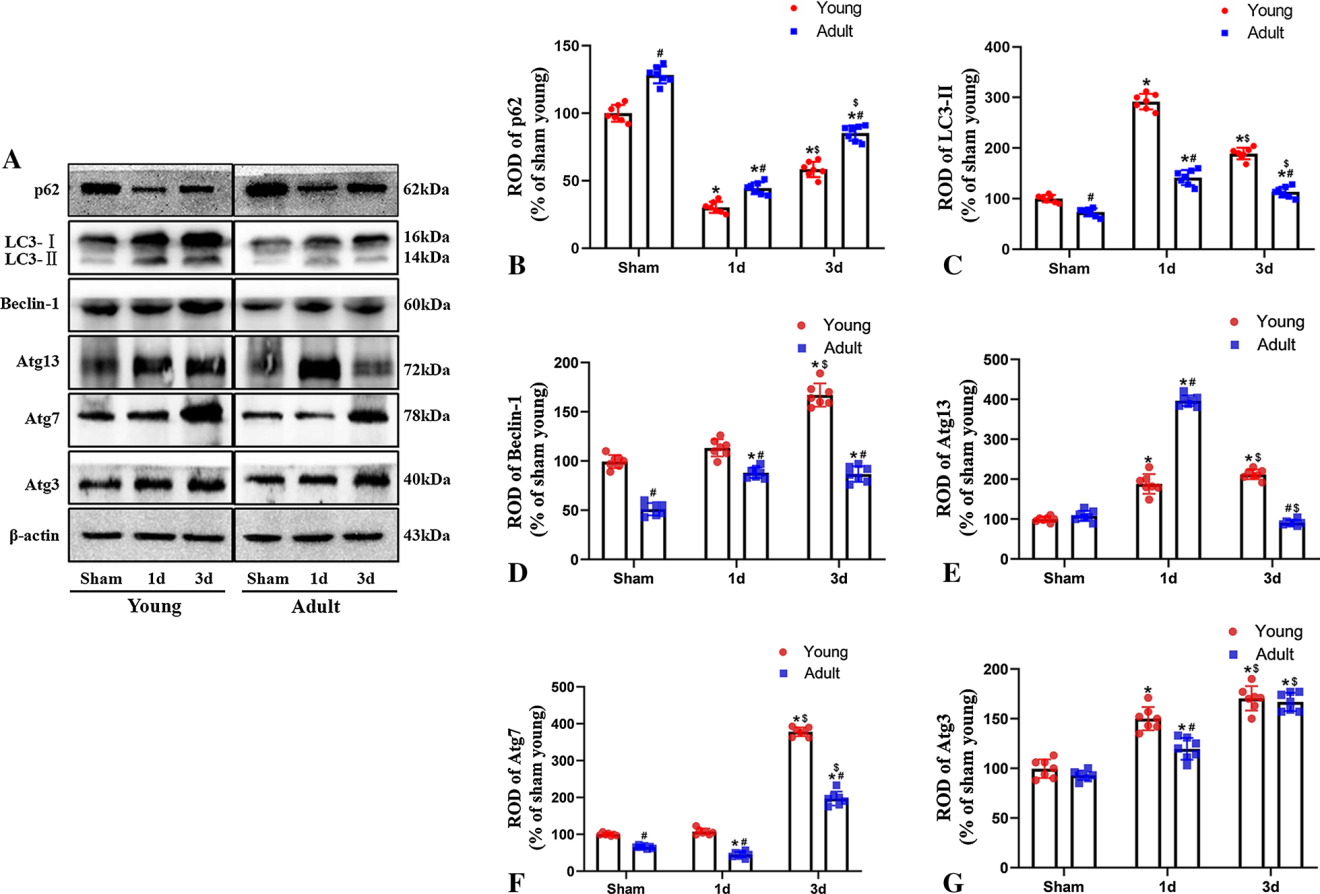
Changes of autophagy-related proteins between young and adult rats after IR. Western blot of autophagy-related proteins (**A**); p62 expression (**B**); LC3-II expression (**C**); Beclin-1 expression (**D**); Atg13 expression (**E**); Atg7 expression (**F**) and Atg3 expression (**G**). (n = 7 per group; ^*^p < 0.05, significantly different from the respectively sham-group; ^#^*p* < 0.05, significantly different from the corresponding young group; ^$^*p* < 0.05, significantly different from the corresponding 1 day group; full-length blots are presented in Additional file 1: Fig. S1). The bars indicate the means ± SEM

After 1 day of IR, the levels of Atg13, an autophagy protein, had increased significantly in adult rats. In young rats, the expression levels of Atg13 had increased significantly after 1 day of IR and then exhibited a significant reduction after 3 days of reperfusion. After 3 days of IR, the expression levels of Atg7 in the cerebral infarction penumbra had increased significantly in both young and adult rats; however, the increase was more pronounced in young rats when compared to adult rats. After 1 and 3 days of IR, the expression levels of Atg3 in the infarcted penumbra increased in both young and adult rats, with expression increasing gradually over time; notably, no significant differences were observed between the young and adult groups. These findings suggest that young rats can improve or maintain the expression levels of Beclin-1, Atg13, and Atg7 after 3 days of IR; moreover, this improved activity enhances LC3-II expression and reduces p62 expression, ultimately reducing neuronal death after IR (Fig. 7). In addition, we found that the levels of LC3-II were significantly lower in the groups receiving 3MA treatment when compared to young and adult rats during the 3 days of IR (Fig. 9).

### Changes in apoptosis-related proteins between young and adult rats after IR

**Fig. 8:**
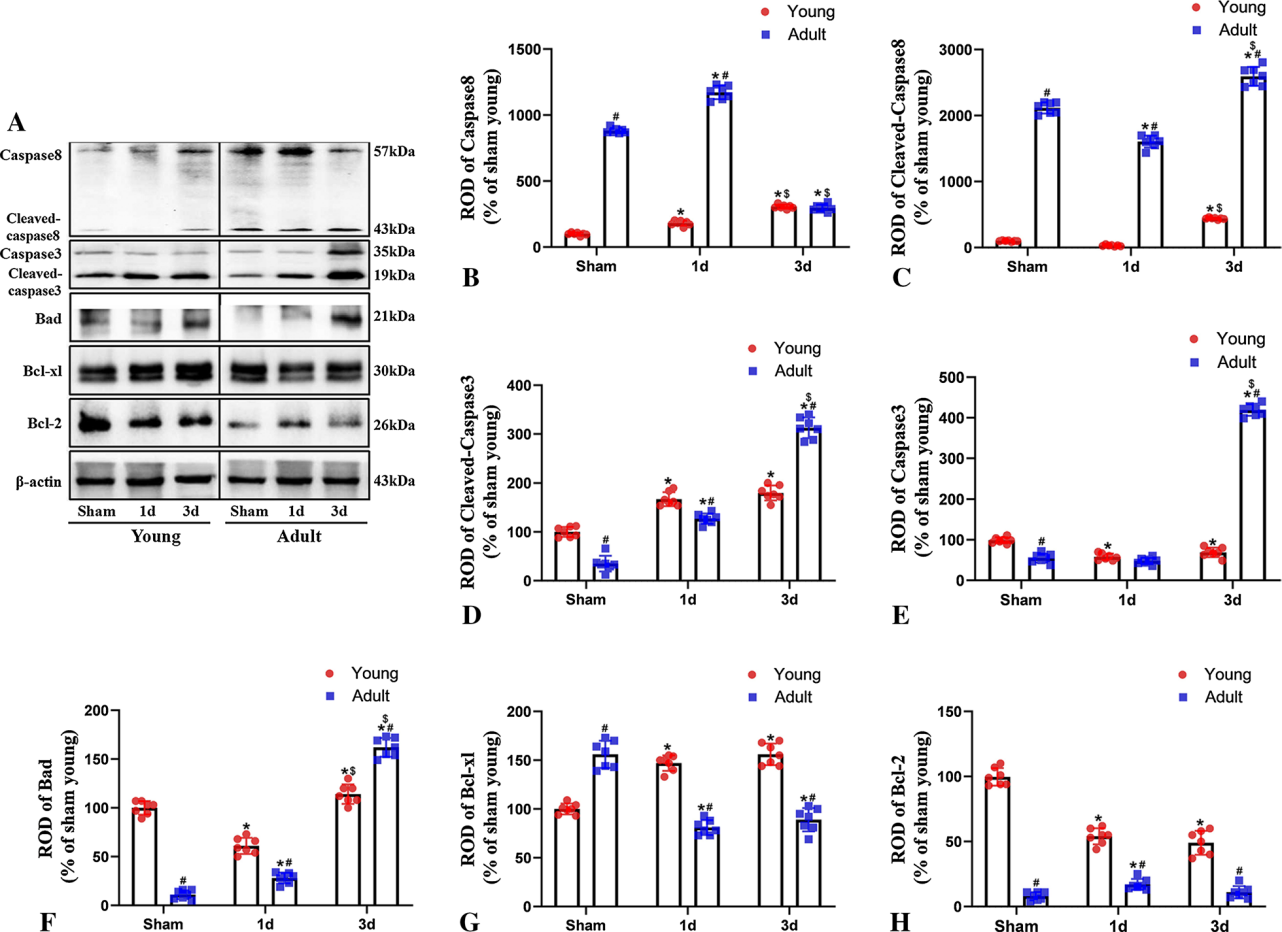
Changes of apoptosis-related proteins between young and adult rats after IR. Western blot analysis of apoptosis-related proteins (**A**); Caspase-8 expression (**B**); Cleaved caspase-8 expression (**C**); Caspase-3 expression (**D**); Cleaved caspase-3expression (**E**); Bad expression (**F**), Bcl-xL expression (**G**) and Bcl-2 expression (**H**). (n = 7 per group; ^*^p < 0.05, significantly different from the respectively sham-group; ^#^*p* < 0.05, significantly different from the corresponding young group; ^$^*p* < 0.05, significantly different from the corresponding 1 day group; full-length blots are presented in Additional file 1: Fig. S2). The bars indicate the means ± SEM

**Fig. 9:**
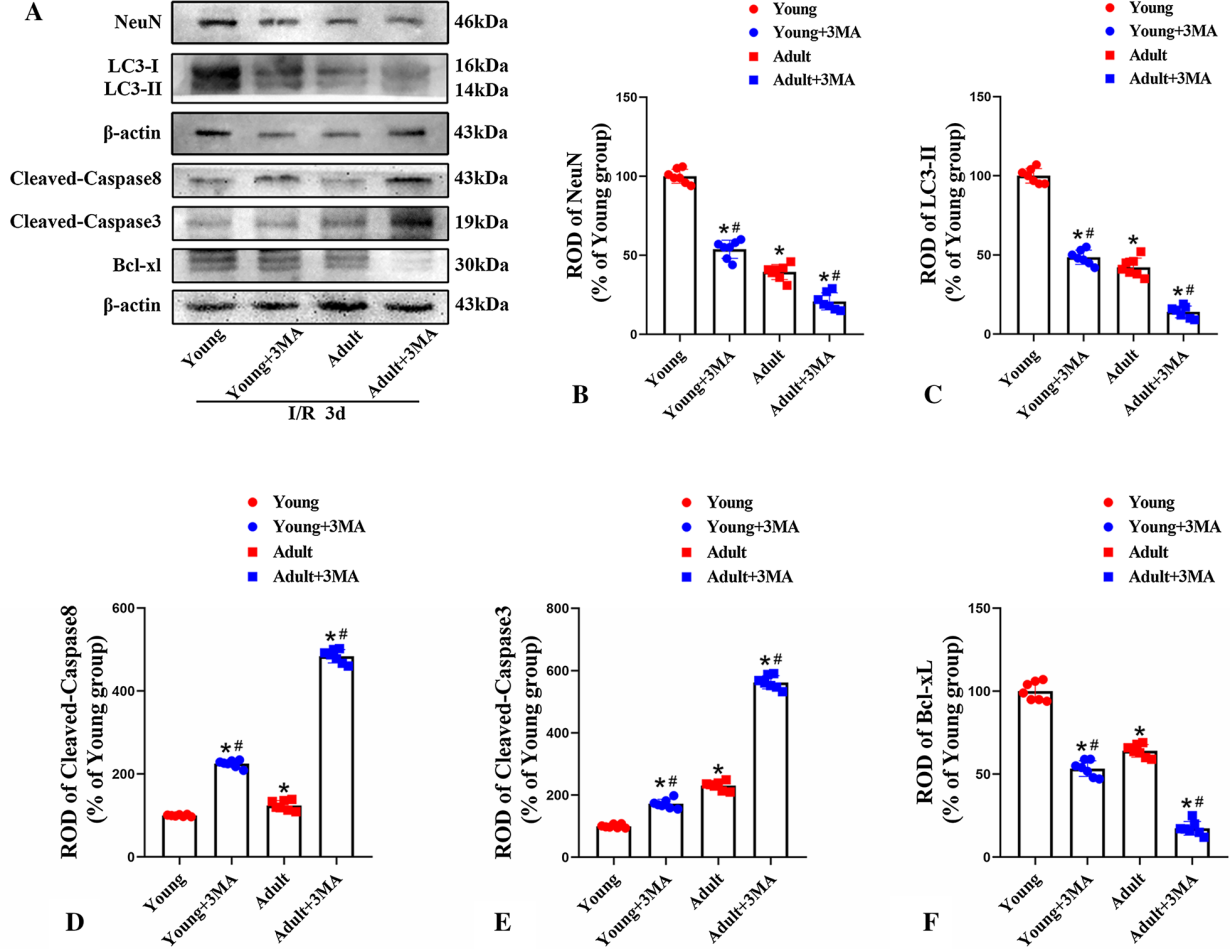
Changes of apoptosis-related proteins between young and adult rats after 3 days of IR by 3MA treatment. Western blot analysis of NeuN expression (**A**, **B**); LC3-II expression (**A**, **C**); Cleaved caspase-8 expression (**A**, **D**); Cleaved caspase-3 expression (**A**, **E**); Bcl-xL expression (**A**, **F**). (n = 7 per group; ^*^p < 0.05, significantly different from the respectively young ischemia-group; ^#^*p* < 0.05, significantly different from the corresponding young or adult ischemia-group; full-length blots are presented in Additional file 1: Fig. S3). The bars indicate the means ± SEM

Finally, we investigated whether the expression levels of apoptosis-related proteins were associated with improvements in cerebral ischemic injury in young rats. Compared to rats in the sham group, rats in the young and adult groups both exhibited significant increases in the levels of Cleaved caspase-8 after 3 days of IR; however, the overall expression levels were higher in the adult group than in the young group. Similarly, following IR, the expression levels of Cleaved caspase-3 slightly increased slightly in young rats and exhibited a more obvious increase in adult rats; these levels gradually increased in accordance with reperfusion time in both groups (Fig. 8). In addition, we found both the levels of Cleaved caspase-3 and Cleaved caspase-8 were significantly higher in the groups receiving 3MA treatment when compared to young and adult rats during the 3 days of IR (Fig. 9).

After 3 days of IR, the expression levels of Bad were higher in both young and adult rats than in rats from the sham group; however, these levels were higher in the adult group than in the young group. After 1 day of IR, the expression levels of Bcl-xL had increased significantly in young rats; expression levels increased gradually in accordance with reperfusion time. In contrast, the expression levels of Bcl-xL decreased in adult rats following IR injury. After 1 day of IR, the expression levels of Bcl-2 increased slightly in adult rats but gradually decreased in accordance with reperfusion time. The expression levels of Bcl-2 in young rats also decreased following IR injury, although the expression levels were higher than in adult rats (Fig. 8). In addition, our present results in Fig. 9 showed that the levels of Bcl-xL were obviously lower in the groups receiving 3MA treatment when compared to young and adult rats during the 3 days of IR (Fig. 9). These findings suggest that neuronal death caused by IR was reduced in young rats due to increased levels of anti-apoptotic proteins and the reduced levels of apoptotic proteins induced by the maintenance of autophagy.

## Discussion

In our previous studies, we reported that the number of neurons decreased significantly in the hippocampus region of mice after MCAO when compared to the sham group [14, 15]. In addition, NeuN immunoreactivity in cells was markedly reduced in the hippocampal CA1 region of gerbils 4 days after IR [3–5, 16]. Consistent with these findings, immunofluorescence assay also confirmed that the number of NeuN positive cells decreased significantly in the infarct areas in MCAO rats [17–19]. In the present study, we employed a rat model of MCAO and revealed that young rats exhibited a better ability to resist cerebral ischemic injury than adult rats. Many previous experimental studies have shown that the degree of stroke-induced ischemic injury depends on the ability of the brain to recover from these injuries and that this ability decreases with age [20]. Thus, there is a close relationship between age-dependent brain self-recovery and the ability of young animals to reduce cerebral focal IR injury [21]. Previous experimental studies by our research group have shown that young gerbils can mitigate the effects of neuronal death after transient global brain ischemia by maintaining the levels of neurotrophic factors, anti-inflammatory factors, and antioxidant enzymes, as well as by reducing the activation and proliferation of glial cells [3–5, 22].

The characteristics of activated glia cells include proliferation and morphological changes; in the central nervous system, glial cells primarily include microglia and astrocytes. Hyperplasia and activation of the glial cells can occur in different neuropathological states and are closely related to the repair of damaged brain tissues or injury to nerve cells. Activated glial cells can secrete large quantities of neurotoxic factors and inflammatory mediators that demonstrate the occurrence of nerve inflammation and cause neuronal death [23]. However, Yan et al. proved that neuronal death in the hippocampal CA1 region after IR was delayed or reduced in young gerbils due to a reduction in glial cell activation [24]. Although the infarct volume of the young rats on the third day of reperfusion with 60 min of ischemia were similar to that observed on the first day with 90 min of ischemia, the level of gliosis was still different. Therefore, although infarct volume has a clear relationship with gliosis, other factors may also be responsible for the differences in infarct volume between young and adult rats. Using a model of focal cerebral ischemia, our present research showed that the proliferation and activation of glial cells in the cerebral infarction penumbra was reduced in young rats and may improve nerve injury and the recovery of nerve function after cerebral ischemia.

Autophagy is a cellular survival mechanism that is essential under physiological and pathological conditions due to its role in maintaining cellular metabolism and homeostasis through the degradation of damaged organelles and macromolecules [25–27]. Because autophagy can remove cellular “garbage” and maintain the dynamic balance between cells, this process is a key factor in neuronal survival [28]. However, the mechanisms underlying the autophagy process after cerebral ischemia remain unclear. Many studies have shown that autophagy induced by ischemic stroke is a neuroprotective process that manifests following stress [8, 29]. Hassanpour M et al. reported that Beclin 1 enhances autophagy, and can also be released from Bcl2 located in the endoplasmic reticulum to form a complex with UVRAG/AMBRA, and trigger the formation of vacuoles by applying Atg7 and Atg10 [30]. In the present study, we showed that the expression levels of LC3-II and Beclin-1 increased in young and adult rats after 1 and 3 days of IR and that the overall expression levels were higher in young rats than in adult rats. This process is controlled by autophagy-related genes. Thus far, 32 Atg genes have been implicated in the induction of autophagy. For example, Atg5 and Atg7 knockout mice exhibit the accumulation of autophagosomes and abnormal organelles, thus resulting in neurodegeneration [31]. In addition, in newborns, mesenchymal stem cells can increase the levels of brain nutrient factors to reduce the activation of mTOR, thereby enhancing autophagy and improving the neurological damage caused by ischemia and hypoxia. Autophagy is known to decrease with age [32], which may be the underlying cause of the strong recovery of cerebral ischemia during early childhood. In the present study, although the expression levels of Atg7 and Atg3 increased after 3 days of IR in both young and adult rats, the expression levels of Atg7 were higher in young rats than in adult rats. Furthermore, after 3 days of IR, the significant increases in Atg13 expression were maintained in young rats but increased and then decreased in adult rats.

Collectively, our present findings suggest that the maintenance or enhancement of Beclin-1, Atg13, and Atg7 expression levels may improve the expression of LC3-II in rats after 3 days of exposure to IR; moreover, these changes may eventually reduce or improve ischemia-induced neuronal death in young rats. Therefore, the enhanced induction of autophagy may be associated with resistance to neuronal damage during cerebral IR in infancy. At the same time, autophagy can be used as a reference to improve the ability to resist cerebral ischemic injury in adults.

Numerous research studies have reported an association between autophagy and apoptosis. Fu et al. found that the apoptosis-related family of caspase proteins can interact with autophagy-related proteins. For example, caspase-3 was shown to inactivate Beclin-1 by cleavage, and then inhibit autophagy and promote apoptosis [33]. Moreover, in T cell studies, Bell et al. found that caspase-8 regulated autophagy, and that autophagy was up-regulated in caspase-8-deficient T cells or T cells that were deficient in proteins related to Fa-associated death domain (FADD) [34]. Some previous studies proved that rapamycin increased autophagy, decreased apoptosis and significantly alleviated brain injury in neonatal rats after hypoxia–ischemia [29]. The neuroprotective mechanism of Macamide B on neonatal hypoxic-ischemic brain damage may be related to the activation of the PI3K/AKT signaling pathway, the enhancement of autophagy, and the reduction of apoptosis induced by hypoxia and ischemia [35]. Chloroquine has been shown to inhibit autophagy and exacerbate neuronal mitochondrial dysfunction and apoptosis in hypoxia rats, thus implying the neuroprotective effects of autophagy in hypoxic-ischemic encephalopathy [36]. In previous studies, agonists of miR-30d-5p were shown to reduce autophagy and increased apoptosis, while inhibitors of miR-30d-5p enhanced autophagy and inhibited apoptosis in rat brains undergoing induced hypoxia–ischemia [37]. The overexpression of HIF-1α was shown to induce significant changes in cellular apoptosis and mitochondrial autophagy in cortical neurons; however, the inhibition of HIF-1α markedly promoted apoptosis and suppressed mitochondrial autophagy [38]. The inhibition of autophagy by 3-MA was previously shown to significantly reduce the expression levels of Beclin-1 while promoting apoptosis; conversely, rapamycin was shown to increase autophagy, increase the expression of Beclin-1, reduce apoptosis, and reduce brain injury [39]. Therefore, we hypothesized that in adult rats, autophagy may be inhibited by the activation of the family of apoptosis-related caspase proteins.

Caspases are known to play important roles in the induction of apoptosis via the external death receptor pathway and pathways involving the mitochondria and endoplasmic reticulum. The caspase proteases can directly cause the disintegration of apoptotic cells and play a central role in mechanisms underlying the apoptosis network. Caspase-3 is a core protease involved in cellular apoptosis; when activated by internal and external factors, caspase-3 immediately initiates a downstream cascade. Thus, cellular apoptosis is inevitable and caspase-3 is often referred to as the “death protease” which can mediate programmed cell death [40]. Caspase-3 is highly expressed in the brain and plays critical roles during neuronal development, cerebral ischemia, and various pathological conditions [41]. Furthermore, the death receptor pathway can activate caspase-8; this activates caspase-3 and induces cellular apoptosis. In the present study, although the levels of Cleaved caspase-8 and Cleaved caspase-3 increased in both young and adult rats after 3 days of IR, the expression levels were higher in the adult group. Furthermore, caspases can directly cleave the Bid precursors of Bcl-2 family members in the cytoplasm to form truncated Bid (tBid), thus triggering the interaction of Bad and Bax to initiate the release of cytochrome C.

Apoptosis signaling is further amplified by the combined actions of the death receptor pathway and the mitochondrial pathway. As a member of the family of Bcl-2 proteins, Bcl-2 is an oncogene that can inhibit apoptosis and effectively reduce the expression and activation of caspase-3. Bcl-xL has inhibitory effects on apoptosis that are similar to those of Bcl-2; other proteins in the Bcl-xL family promote apoptosis, including Bad and Bax [42]. Knockout of the caspase-3 gene significantly improves ischemia-induced neuronal death in both in vivo and in vitro models; moreover, the activation of caspase-8 causes sustained neuronal damage following cerebral blood reperfusion [43, 44]. Our present findings demonstrate that, as an intermediate trigger in the promotion of apoptosis, the expression of Bad increased in both young and adult rats after IR, with significantly higher expression levels in adult rats. However, the levels of Bcl-xL expression gradually increased after 3 days of cerebral IR in young rats; in adult rats, the levels of Bcl-xL expression gradually decreased. Moreover, Bcl-2 expression levels were always higher in young rats than in adult rats. Collectively, these results indicate that increases in the levels of anti-apoptotic proteins and reductions in the levels of apoptotic proteins were closely related to the resistance to ischemia in young animals.

## Conclusion

In the present study, we demonstrated that the reduced infarct volumes and reductions in neuronal death observed after focal ischemic stroke in young rats were closely related to the maintenance and enhancement of autophagy levels in nerve cells, as well as the regulation of apoptotic factor expression levels and the inhibition of glial activation. In addition, our results showed that the expression levels of proteins related to autophagy were negatively correlated with the activation of apoptosis-related caspase proteins. These factors may represent the mechanism of action by which young animals were more resistant to ischemic injury.

## Supplementary Information

**Additional file 1:** Original blots of autophagy-related proteins and apoptosis-related proteins between young and adult rats after IR.

**Additional file 2:** Neurological score between the young and adult groups (1 and 3 days of reperfusion after 30, 60 and 90 min of ischemia).

## Data Availability

The datasets generated and/or analysed during the current study are not publicly available due to my work privacy institutional commitments but are available from the corresponding author Bing Chun Yan (bcyan@yzu.edu.cn) on reasonable request.
