# Supplementary material for: Enhanced autophagy interacting proteins negatively correlated with the activation of apoptosis-related caspase family proteins after focal ischemic stroke of young rats
Source: BMC Neurosci. 2022 Sep 28;23:55. doi: 10.1186/s12868-022-00740-w (PMC9516802; doi:10.1186/s12868-022-00740-w)
Supplement: Supplementary file 1 — Additional file 1: Original blots of autophagy-related proteins and apoptosis-related proteins between young and adult rats after IR. [file 12868_2022_740_MOESM1_ESM.docx]

Additional file

for

**Enhanced autophagy interacting proteins negatively correlated with the activation of apoptosis-related caspase family proteins after focal ischemic stroke of young rats**

Jie Wang^1,2,†^, Zihao Xia^1,†^ , Peng Sheng ^1^ , Mengmeng Shen^1^, Lidong Ding^3^, Dezhi Liu^4,^*, Bing Chun Yan^1,^*

*^1^Medical College, Institute of Translational Medicine, Department of Neurology, Affiliated Hospital of Yangzhou University, Jiangsu Key Laboratory of Integrated Traditional Chinese and Western Medicine for Prevention and Treatment of Senile Diseases, The Key Laboratory of Syndrome Differentiation and Treatment of Gastric Cancer of the State Administration of Traditional Chinese Medicine, Yangzhou University, Yangzhou, 225001, PR China; Jiangsu Key Laboratory of Zoonosis, Jiangsu Co-Innovation Center for Prevention and Control of Important Animal Infectious Diseases and Zoonoses, Yangzhou, 225009, PR China*

*^2^Department of Peripheral Vascular Surgery, Shuguang Hospital Affiliated to Shanghai University of Traditional Chinese Medicine, Shanghai, 201203, PR China*

*^3^Department of Neurology, Taizhou Second People’s Hospital, Taizhou 225500, PR China*

*^4^Department of Neurology, Shuguang Hospital Affiliated to Shanghai University of Traditional Chinese Medicine, Shanghai, 201203, PR China*

^†^ Jie Wang and Zihao Xia contribute equally to this article.

* Corresponding authors:

Professor Bing Chun Yan, E-mail: bcyan@yzu.edu.cn

Professor Dezhi Liu, E-Mail: yzldz@126.com

**
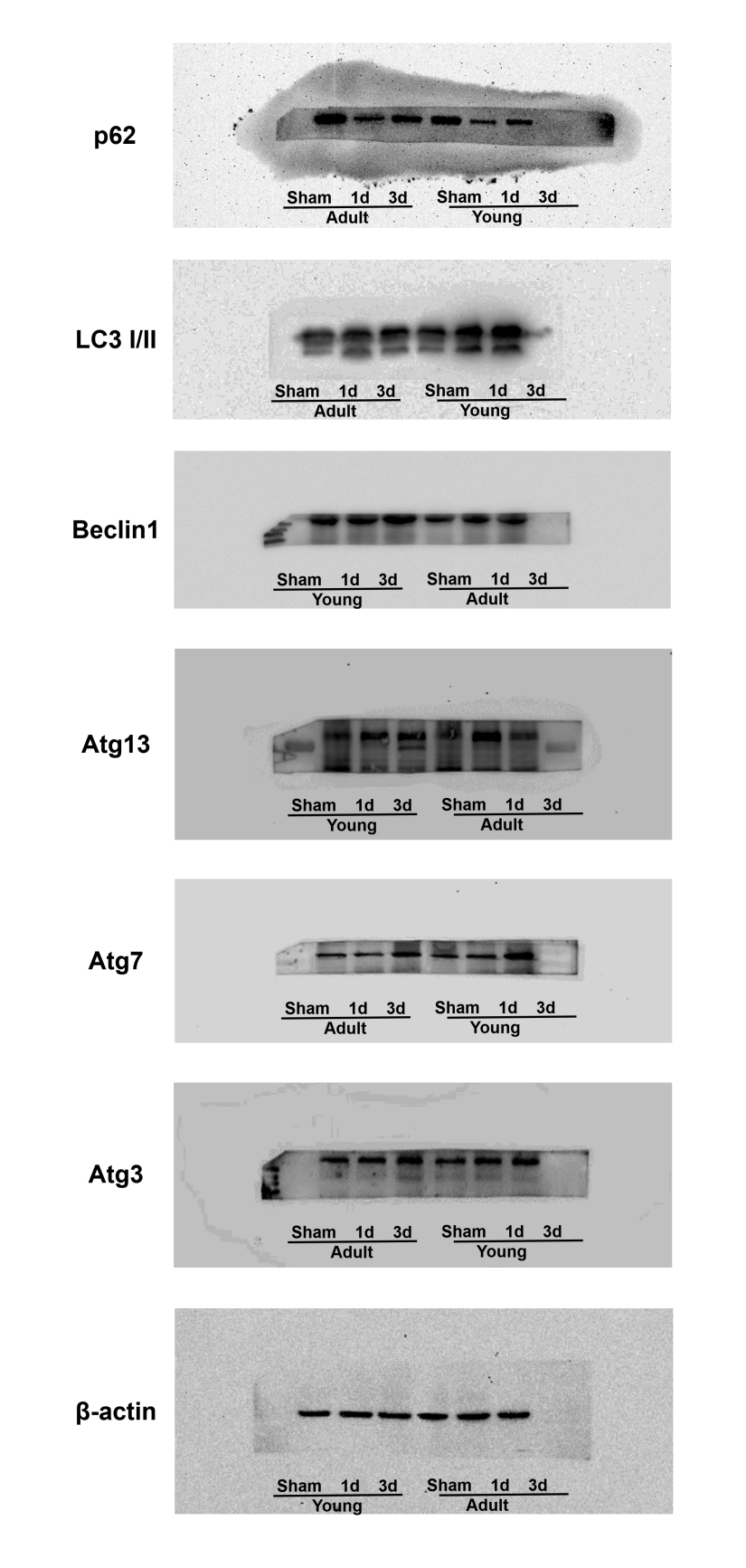
**

**Figure S1.** Original blots of autophagy-related proteins between young and adult rats after IR. (The membranes were cut into proper bands prior to hybridisation with antibodies).


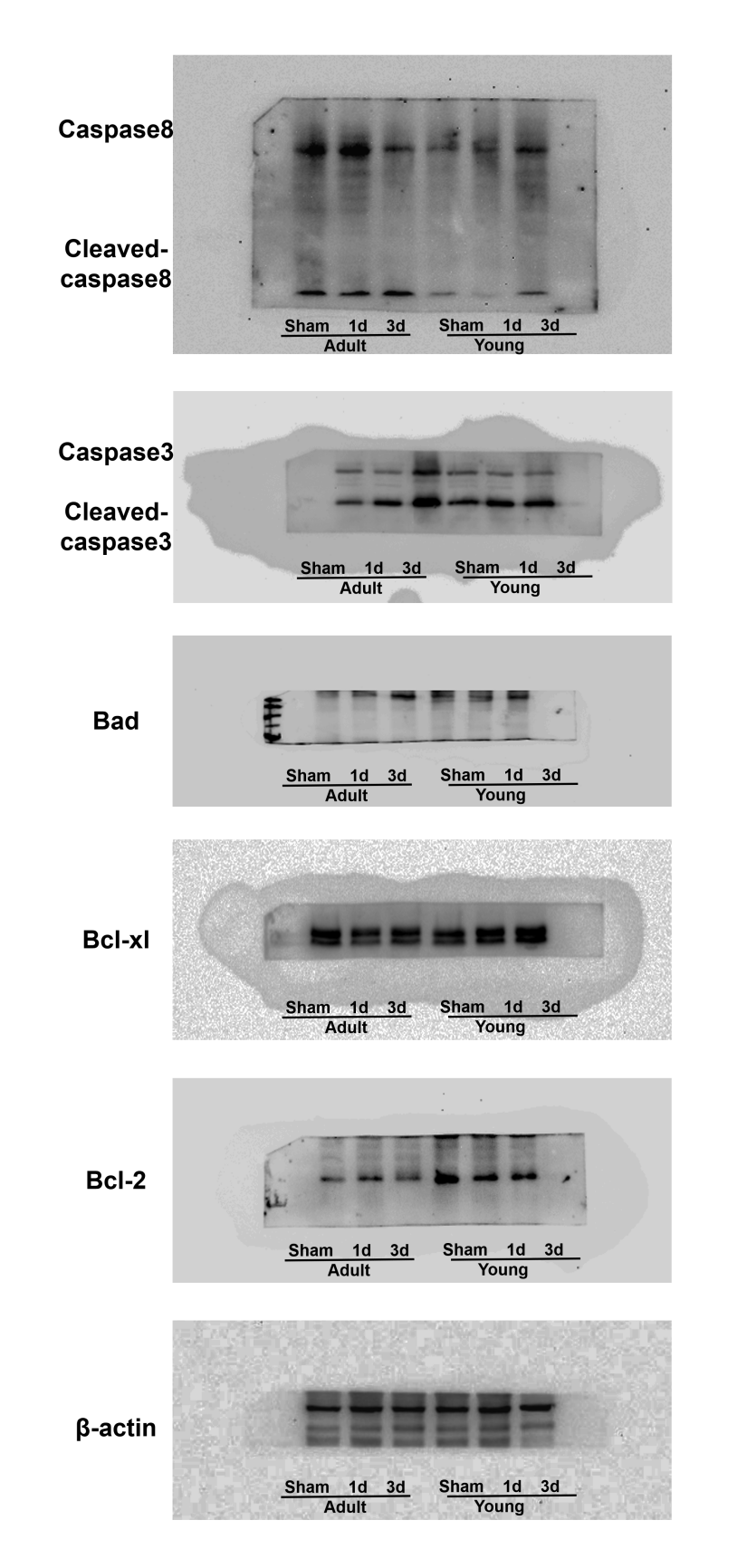


**Figure S2.** Original blots of apoptosis-related proteins between young and adult rats after IR. (The membranes were cut into proper bands prior to hybridisation with antibodies).


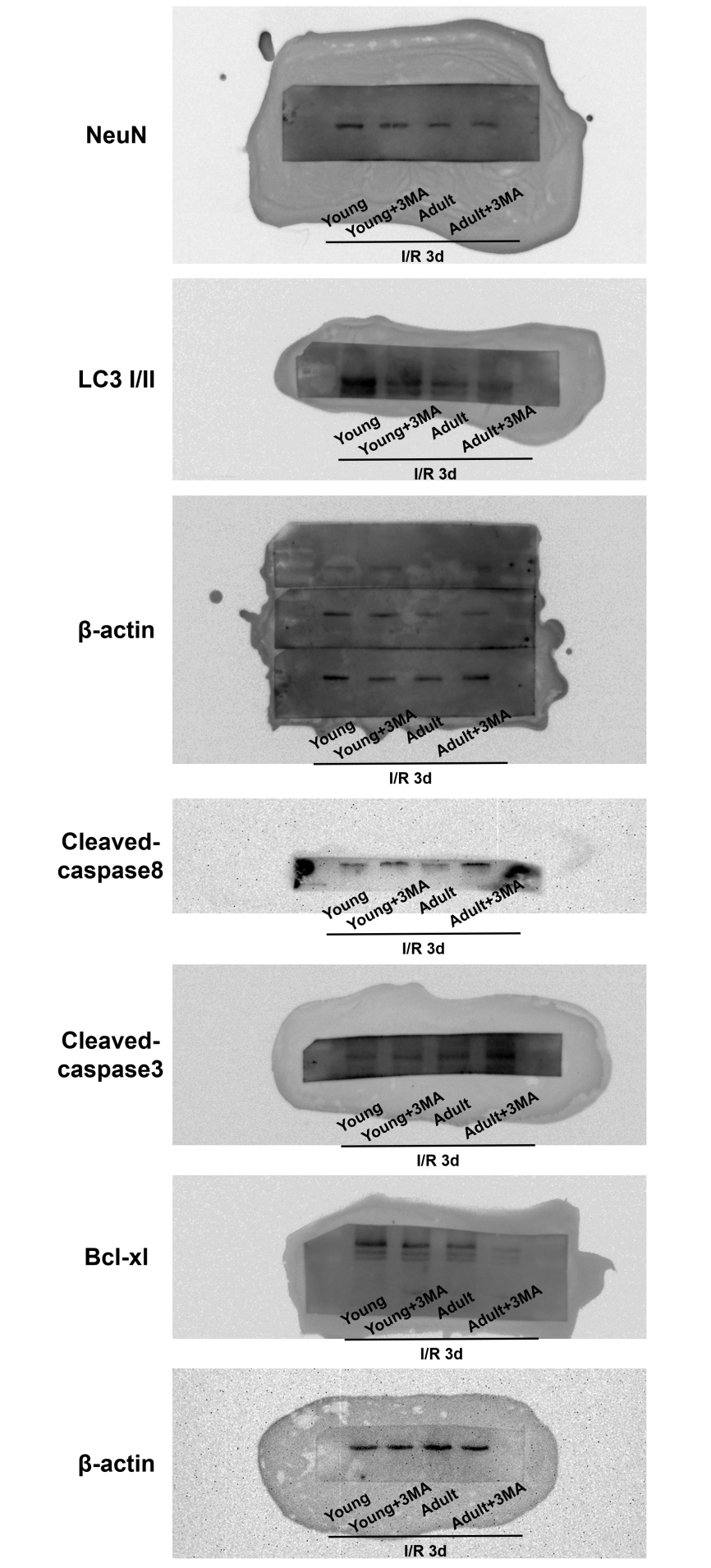


**Figure S3.** Original blots of apoptosis-related proteins between young and adult rats after 3 days of IR by 3MA treatment. (The membranes were cut into proper bands prior to hybridisation with antibodies).
